# Favorable fatty acid composition in adipose tissue in healthy Iraqi- compared to Swedish-born men — a pilot study using MRI assessment

**DOI:** 10.1080/21623945.2022.2042963

**Published:** 2022-03-16

**Authors:** Lena Trinh, Karin G Stenkula, Lars E Olsson, Jonas Svensson, Pernilla Peterson, Louise Bennet, Sven Månsson

**Affiliations:** a Medical Radiation Physics, Department of Translational Medicine, Lund University, Malmö, Sweden; b Department of Experimental Medical Sciences, Lund University, Lund, Sweden; c Hematology, Oncology and Radiation Physics, Skåne University Hospital, Malmö, Sweden; d Medical Imaging and Physiology, Skåne University Hospital, Lund, Sweden; e Department of Clinical Sciences, Lund University, Malmö, Sweden; f Clinical Research and Trial Centre, Lund University Hospital, Lund, Sweden

**Keywords:** Adipose tissue composition, migration, water-fat imaging, cardiometabolic disease, MRI

## Abstract

Middle Eastern immigrants are at high-risk for insulin resistance. Fatty acid composition (FAC) plays an important role in the development of insulin resistance but has not been investigated in people of Middle Eastern ancestry. Here, the aim was to assess the FAC in visceral and subcutaneous adipose tissue (VAT and SAT) in healthy Iraqi- and Swedish-born men using a magnetic resonance imaging (MRI) method.This case-control study included 23 Iraqi- and 15 Swedish-born middle-aged men, without cardiometabolic disease. Using multi-echo MRI of the abdomen, the fractions of saturated, monounsaturated, and polyunsaturated fatty acids (fSFA, fMUFA, and fPUFA) were estimated in VAT and SAT. SAT was further analyzed in deep and superficial compartments (dSAT and sSAT).

In all depots, fPUFA was significantly higher and fSFA significantly lower in Iraqi men, independently of age and BMI. In both Iraqi- and Swedish-born men, higher fPUFA and lower fMUFA were found in sSAT vs. dSAT. Among Iraqi men only, higher fPUFA and lower fMUFA were found in SAT vs. VAT.Iraqi-born men presented a more favorable abdominal FAC compared to Swedish-born men. This MRI method also revealed different FACs in different abdominal depots. Our results may reflect a beneficial FAC in Middle Eastern immigrants.

## Introduction

Middle East immigrants represent the largest immigrant population in Europe and Sweden today [1]. Register-based data show that first-generation immigrants have twice the risk of developing type 2 diabetes compared to the Swedish-born population [2]. Further, a population-based study including over 2000 people born in Iraq or Sweden with a high representativeness has shown lower blood pressure and better kidney function, despite higher prevalence of insulin resistance and the metabolic syndrome [3,4]

In general, the association between accumulation of fat within ectopic and visceral depots and risk of various metabolic diseases such as insulin resistance and type 2 diabetes has been well documented [5–7]. However, not only the amount and location of the accumulated fat are of interest, but also the fatty acid composition (FAC), i.e., the proportions of saturated, monounsaturated, and polyunsaturated fatty acids (SFA, MUFA, and PUFA, respectively) may play a role. For instance, studies have shown that the FAC of dietary fat, which in turn affects the FAC of adipose tissue [8,9], has an impact on the risk of developing type 2 diabetes [10,11], hypertension [12], and cardiovascular disease (CVD) [13,14]. Dietary SFA has been identified as a risk factor for the development of CVD [13,14].

Furthermore, the FAC of adipose tissue seems to depend on its location in the body. Previous studies have reported a higher relative amount of SFA in the visceral adipose tissue (VAT) compared to the subcutaneous adipose tissue (SAT) [15]. Similarly, it has been shown that the FAC of deep and superficial SAT (dSAT and sSAT, respectively) differ and that the FAC of dSAT may have a greater association with disease risk [16,17]. The assessment of the FAC of various adipose tissue depots might therefore be of interest in an attempt to better understand the role of adipose tissue FAC in metabolic and CVD.

The golden standard method of measuring adipose tissue FAC is gas chromatography analysis of biopsy samples, an invasive method that assesses FAC only at a single position. Non-invasive alternatives for FAC quantification have been introduced, first through magnetic resonance spectroscopy (MRS) [18,19], and more recently by magnetic resonance imaging (MRI) [20–23]. While single-voxel MRS also measures FAC at a single position, the MRI approach offers the possibility to examine a larger volume in a single measurement. Thus, MRI allows simultaneous assessment of FAC of several adipose tissue depots or larger body parts, and can also be used to examine fat located deep within the body, for example, visceral adipose tissue [24], bone marrow [25], or other organs [26]. Studies using the MRI-based method have so far mainly been exploratory [20–23]. While the number of studies where the MRI method has been used in larger groups of volunteers or patients is limited [24,26,27], the method has shown promising results in studies where gas chromatography has been used as a reference method [24,27].

Although the knowledge of how fat contributes to the development of metabolic syndromes has increased over the years, the exact relationship between FAC in adipose tissue and metabolic syndrome and CVD are not yet fully understood.

Since the Iraqi-born population is one of the largest immigrant groups in Sweden, with a high risk of type 2 diabetes, it is important to obtain more accurate estimations of risk factors of this population. From a clinical perspective, Iraqi-born subjects with normal weight and blood pressure may be considered having a low risk for CVD, but traditional cardiovascular risk factors, such as BMI and blood pressure might not reflect cardiovascular risk equally across Middle Eastern and European ethnicities [28]. The use of inaccurate risk profiles may result in an underestimation of the CVD hazard. Therefore, for better understanding of the role of adipose tissue for the CVD risk across Middle Eastern and European ethnicities, further investigations of adipose tissue distribution and FAC across ethnicities are needed. The aim of this study was thus to investigate and compare the FAC of abdominal adipose tissue of non-obese healthy men, born in Iraq to those born in Sweden, using the MRI-based method.

## Methods

### Subjects

**Figure 1.**
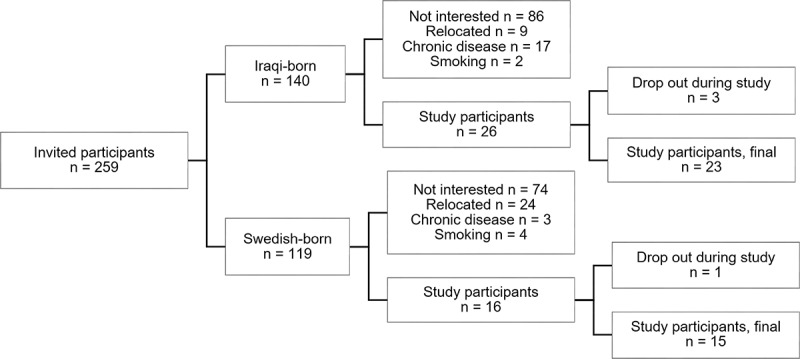
Flowchart for invited and included study participants.

A total of 38 healthy males were included in this study, of which 23 were Iraqi-born and 15 were Swedish-born residents in the city of Malmö, Sweden. The participants were recruited from the MEDIM cohort consisting of over 2100 individuals born in Iraq or Sweden [29]. Only healthy, non-smoking, non-obese (BMI < 30 kg/m^2^) men without cardiovascular risk factors or established cardiometabolic disease were invited to participate. A flow chart over invited and included study participants is shown in Figure 1. Participation was voluntary at all stages and all subjects signed an informed consent before participation. Approval for the study was granted from the Ethical Review Board of Lund University (2015/507).

### Anthropometric measures and blood sampling

Anthropometrics including height, waist–hip ratio, systolic and diastolic blood pressure, and body mass index (BMI) were assessed. Fasting blood samples were collected and analysed. Blood glucose was measured immediately after sampling in capillary whole blood (HemoCue AB, Ängelholm, Sweden) [30]. Total cholesterol and plasma triglycerides (p-TG) were assessed by enzymatic methods (Bayer Diagnostics) [31], High-density lipoprotein cholesterol (p-HDL) and low-density lipoprotein cholesterol (p-LDL) were measured enzymatically using Friedewald’s method [32]. Fat mass and fat-% were assessed by bioelectrical impedance analysis (BIA) (Tanita Pro, Tanita Europe BV, The Netherlands). BIA estimates the body composition by sending a weak electrical current through the body and calculating its impedance. The accuracy of the Tanita system is within ±5% of the gold standard methods underwater weighing and DEXA [33]. The characteristics of the subjects are summarized in Table 1.

**Table 1.** Summary of age, anthropometric measures, blood pressure, dietary habits and blood sample analyses for the subject groups. Values are presented as mean (range), except for the dietary habits. p-values for the differences between the groups were calculated using an unpaired two-sample t-test

	Iraqi-born	Swedish-born	p-value (difference)
Age (years)	49.2 (36–69)	51.8 (37–71)	0.5
BMI (kg/m^2^)	26.9 (23.9–29.8)	25.7 (22.7–29.2)	0.07
Systolic blood pressure (mmHg)	115 (98–131)	128 (102–160)	0.01
Diastolic blood pressure (mmHg)	67 (58–79)	72 (57–89)	0.1
Waist-hip ratio	0.96 (0.87–1.05)	0.94 (0.84–1.06)	0.26
Diet score (points)	8.3 (2–15)	8.8 (4–13)	0.67
Proportion with unhealthy dietary habits^b^ (%)	57.7% (n = 15)	56.3% (n = 9)	0.89 ^a^
Prefer butter from oil in cooking (%)	3.8% (n = 1)	50% (n = 8)	<0.001
Consumption of food twice weekly or more, containing animal fat (%)	19.2% (n = 5)	68.8% (n = 11)	0.002^a^
Triglyceride, blood (mmol/L)	1.5 (0.5–3.2)	1.0 (0.6–2.0)	0.06
Fat-%	25.0% (20.4–31.2)	22.8% (8.6–28.7)	0.1
Fat mass (kg)	20.0 (15–27)	19.0 (6.7–26.9)	0.5
Blood glucose (mmol/L)	5.7 (4.9–7.2)	5.8 (5.2–6.5)	0.7
Cholesterol (mmol/L)	4.9 (1.6–6.8)	4.9 (3.3–5.8)	0.9
High density Lipoproteins (HDL) (mmol/L)	1.1 (0.8–1.6)	1.5 (0.9–2.4)	0.002
Low density lipoproteins (LDL) (mmol/L)	3.7 (2.5–5.7)	3.4 (2.0–4.3)	0.3

### Dietary assessment

All participants filled out the National Board of Health and Welfare validated multiple-choice questionnaires on healthy diet habits capturing the frequency of consumption of 1) vegetables (fresh or frozen), 2) fruit/berries (fresh, frozen or juice), 3) fish or seafood, 4) pastries, candy, soda and 5) breakfast [34]. Each question gave 0 to 3 points depending on the frequency of the habit, with higher points reflecting healthier habits. The maximum score was 15 points and those with less than nine points were considered having unhealthy eating habits [34].

Participants also answered questions if they preferred butter or oil in cooking and how often they consumed food twice or more on weekly bases containing animal fat.

### MRI acquisition

Axial monopolar multi-echo gradient echo 2D images of the abdomen were acquired using a 3 T MRI scanner (Tim Trio, Siemens Healthineers, Erlangen, Germany) with a 6-element body matrix flex coil and a 24-element spine array. Ten image slices were acquired, centred at the L3-L4 disc. The following parameters were used: number of echoes = 12, TE1/ΔTE = 1.13/1.56 ms, TR = 200 ms, matrix size = 128x96, FOV = 380x285x8.5 mm^3^, flip angle = 10°, and bandwidth = 1628 Hz/pixel. The images were collected during one breath-hold with a scanning time of 20 sec.

### Data analysis

The MRI-based approach used to assess the FAC in this study is based on the theoretical expressions previously suggested by Hamilton et al. [18] where the triglyceride molecules are described by the number of double bonds (*ndb*), the number of methylene-interrupted double bonds (*nmidb*), and chain length. A brief overview of the method will be given here as the general approach has been described in detail previously [22,27]. In this study, the theoretical expressions suggested by Hamilton et al. were modified by using a fixed chain length value [35], as opposed to estimating the chain length as a free parameter. This reduces the number of unknown parameters, thus increasing the robustness of the algorithm [35]. A fixed chain length was also motivated by the small interpersonal variation of this variable reported in previous studies of human adipose tissue [9,36]. Based on previously published results obtained from gas chromatography analysis of SAT, the chain length was set to 17.3 [27].

**Figure 2.**
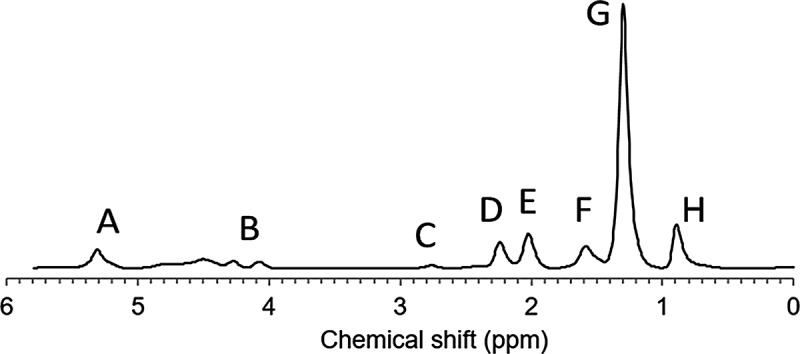
An example MR spectrum of subcutaneous adipose tissue with the corresponding fat resonance groups (a-h).

The acquired MRI signal *S* at echo time *t* can be described by

(1a) $$S\left(t\right)=\left(W+Ff\overset{M}{\underset{m}{\sum }}\alpha_{m}E_{m}\left(t\right)\right)e^{\Psi t},$$

where *W* and *F* are the water and fat signal amplitudes, $\alpha_{m}$ is the amplitude of resonance group *m* (Figure 2 and Table 2), $f=1/\sum \alpha_{m}$ is a normalization factor, and $\Psi =i2\pi \psi -R_{2}^{*}$ is a complex field map [37]. Using the expressions in Hamilton et al. [18], Eq. (1a) can be rewritten as

(1b) $$S\left(t\right)=\left(W+Ff\left(P_{F}\left(t\right)+P_{ndb}\left(t\right)ndb+P_{nmidb}\left(t\right)nmidb\right)\right)e^{\Psi t}$$

**Table 2.** An eight-resonance fat model, with resonance groups (A-H) and the corresponding chemical shifts and amplitudes. The amplitudes are modified versions of the ones introduced by Hamilton et al. [18] to implement a fixed chain length value of 17.3 [27]. ndb = number of double bonds, nmidb = number of methylene-interrupted double bonds

Resonance group *m*	Chemical shift (ppm)	Assignment	Theoretical amplitudes $\alpha_{m}$
A	5.28	-C**H**= C**H**- -C**H**-O-CO-	2*ndb* 1
Water	4.7	**H**_2_O	–
B	4.22	-C**H**_2_-O-CO-	4
C	2.75	-CH = CH-C**H**_2_-CH = CH-	2*nmidb*
D	2.25	-CO-C**H**_2_-CH_2_-	6
E	2.02	-C**H**_2_-CH = CH-C**H**_2_-	4(*ndb – nmidb*)
F	1.57	-CO-CH_2_-C**H**_2_-	6
G	1.30	-(C**H**_2_)_n_-	79.8–8*ndb* + 2*nmidb*
H	0.90	-(CH_2_)_n_-C**H**_3_	9

**Table 3.** Median of the estimated fractions of saturated, monounsaturated, and polyunsaturated fatty acids (f_SFA_, f_MUFA_, f_PUFA_), number of double bonds (ndb), and number of methylene-interrupted double bonds (nmidb) with corresponding interquartile ranges, in SAT, VAT, dSAT and sSAT. The differences between the Iraqi-born and Swedish-born men and the corresponding p-values are also presented

		Swedish-born	Iraqi-born	Difference	
Depot		Median (interquartile range)	Median (range)	*f*_Iraqi_ – *f*_Swede_	p-value
SAT	*f*_SFA_	0.350 (0.332–0.369)	0.308 (0.284–0.324)	−0.042	<0.001
	*f*_MUFA_	0.569 (0.539–0.596)	0.540 (0.507–0.554)	−0.029	0.02
	*f*_PUFA_	0.085 (0.066–0.111)	0.160 (0.143–0.186)	0.075	<0.001
	*ndb*	2.24 (2.08–2.33)	2.56 (2.52–2.63)	0.33	<0.001
	*nmidb*	0.26 (0.20–0.33)	0.48 (0.43–0.56)	0.22	<0.001
VAT	*f*_SFA_	0.340 (0.322–0.374)	0.304 (0.286–0.315)	−0.036	<0.001
	*f*_MUFA_	0.588 (0.552–0.613)	0.568 (0.525–0.582)	−0.021	0.09
	*f*_PUFA_	0.073 (0.059–0.084)	0.131 (0.116–0.153)	0.058	<0.001
	*ndb*	2.20 (2.09–2.27)	2.49 (2.42–2.61)	0.29	<0.001
	*nmidb*	0.22 (0.18–0.25)	0.39 (0.35–0.46)	0.17	<0.001
dSAT	*f*_SFA_	0.352 (0.332–0.378)	0.299 (0.291–0.320)	−0.053	<0.001
	*f*_MUFA_	0.581 (0.537–0.599)	0.548 (0.524–0.559)	−0.034	0.09
	*f*_PUFA_	0.074 (0.062–0.088)	0.148 (0.141–0.176)	0.074	<0.001
	*ndb*	2.14 (2.03–0.2.26)	2.56 (2.49–2.63)	0.42	0.004
	*nmidb*	0.22 (0.19–0.26)	0.44 (0.42–0.53)	0.22	0.004
sSAT	*f*_SFA_	0.348 (0.327–0.367)	0.302 (0.280–0.312)	−0.046	<0.001
	*f*_MUFA_	0.558 (0.515–0.585)	0.512 (0.484–0.541)	−0.046	0.04
	*f*_PUFA_	0.102 (0.070–0.131)	0.179 (0.161–0.226)	0.077	<0.001
	*ndb*	2.27 (2.16–2.34)	2.64 (2.56–2.75)	0.37	0.004
	*nmidb*	0.31 (0.21–0.39)	0.54 (0.58–0.68)	0.23	0.01

where *P_F_,P_ndb_*, and *P_nmidb_* are the coefficients related to the variables *F, ndb*, and *nmidb*, respectively, obtained from the theoretical amplitude expressions for each resonance, given in Table 2 for an eight-resonance fat model:

$$P_{F}\left(t\right)=E_{A}+4E_{B}+6E_{D}+6E_{F}+79.8E_{G}+9E_{H}$$

$$P_{ndb}\left(t\right)=2E_{A}+4E_{E}-8E_{G}$$

$$P_{nmidb}\left(t\right)=\;2E_{C}-4E_{E}+2E_{G}$$

$E_{m}=e^{i\omega_{m}t}$, where $\omega_{m}$ is the angular frequency of fat resonance *m*. An iterative least-squares reconstruction algorithm with a joint estimation of *W, F, ndb, nmidb*, and Ψ was then used to solve Eq. 1b after a conversion to matrix form [22,37]. All calculations were conducted using MATLAB R2019b (MathWorks, Natick, MA, USA).

Assuming that fatty acids have at most two double bonds, the fractions of SFA (*f_SFA_*), MUFA (*f_MUFA_*), and PUFA (*f_PUFA_*), respectively, can be calculated from the estimated *ndb* and *nmidb* as follows [19,22,38]:

$$f_{SFA}=1-\frac{ndb-nmidb}{3}$$

$$f_{MUFA}=\frac{ndb-2\cdot nmidb}{3}$$

$$f_{PUFA}=\frac{nmidb}{3}$$

Fat fraction maps were obtained by calculating the ratio *F*/(*W* + *F*). Estimations of *f_SFA_,f_MUFA_,f_PUFA_*, and fat fraction were calculated voxel-by-voxel, creating FAC maps.

**Figure 3.**
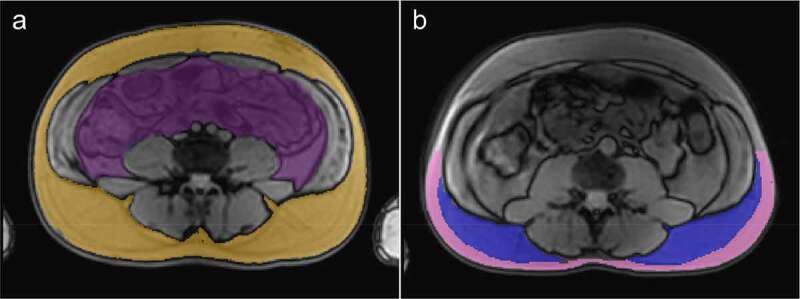
A) Example of a subcutaneous mask (Orange), outlined using a region-growing algorithm [39], and a visceral depot mask (purple), outlined manually to avoid the spinal area. b) Example of sSAT (pink) and dSAT (blue) masks, manually separated using SAT ROIs. Only posterior adipose tissue was included in the estimation of dSAT and sSAT FAC due to difficulties to separate anterior dSAT and sSAT.

To separate the subcutaneous and the visceral depot, a first approximation of the SAT was outlined using a region-growing algorithm [39], whereas the abdominal cavity was manually delineated to avoid the spinal area (Figure 3). All depot delineations were visually inspected before used in further analysis. In images where the Scarpa’s fascia could be clearly distinguished, the subcutaneous depot was further manually divided into a deep and a superficial SAT (dSAT and sSAT, respectively). This resulted in a total of 10 Swedish-born and 17 Iraqi-born men where the dSAT and sSAT could be separated from each other. For this separation, only SAT within the lower half of the images was used, due to the difficulties to separate sSAT and dSAT in the anterior area. The final regions-of-interest (ROIs) were then defined as the voxels with fat fractions higher than 0.9, and T2* longer than 20 ms within each outlined depot (VAT, SAT, sSAT, and dSAT). The FAC was evaluated as the mean value within the respective ROIs.

### Statistical analysis

The difference in *ndb, nmidb, f_SFA_,f_MUFA_*, and *f_PUFA_* between the Swedish-born and the Iraqi-born men were statistically tested using a Wilcoxon rank-sum test. To compare the FAC of VAT and SAT, and of sSAT and dSAT, Wilcoxon signed-rank test were conducted. Differences in age, anthropometric measures, blood pressure, blood sample analyses, and dietary habits were tested with an unpaired two-sample t-test. Tests with p-values less than 0.05 were considered significant. A multiple linear regression model was used to investigate relationships between the parameters in Table 1 and the FAC parameters. All FAC parameters and all continuous-valued parameters in Table 1 were normally distributed according to the Kolmogorov–Smirnov test. All statistical tests were carried out using MATLAB and SPSS.

## Results

A considerably lower proportion of the Iraqi-born men reported that they preferred to use butter rather than oil when preparing food, or that they regularly consumed food containing animal butter, indicating a lower consumption of saturated fats in Iraqi men (Table 1). Otherwise, Iraqi-born men reported as healthy food habits as Swedish-born men regarding the National Board of Health and Welfare indicator questions of healthy eating habits [34]. Eighty percentage of the Iraqi-born men were overweight (BMI > 25 kg/m^2^), vs. 62% of the Swedish-born. The Iraqi-born men also had significantly lower systolic blood pressure than the Swedish-born (115 vs. 128 mmHg, p = 0.01).

Significantly lower *f_SFA_* and *f_MUFA_*, and significantly higher *f_PUFA_* were found in the SAT of the Iraqi-born men, compared to the Swedish-born men (Figure 5 and Table 3). Very similar results were found also when comparing dSAT and sSAT separately, except for the difference of *f*_MUFA_ in dSAT being non-significant. In the VAT depot, significantly lower *f_SFA_* and significantly higher *f_PUFA_* were found in Iraqi-born men. Although also *f_MUFA_* tended to be lower in the visceral depot of Iraqi-born compared to Swedish-born men, the difference was not significant.

Using the multiple linear regression model, diet score and consumption of animal fat were positively associated with *f*_SFA_, resulting in moderate correlations (SAT: R = 0.68, p = 0.0001, VAT: R = 0.54, p = 0.003). Unhealthy eating habits were positively associated with *f*_MUFA_, but resulted in weaker correlations (SAT: R = 0.34, p = 0.039, VAT: R = 0.43, p = 0.010). Consumption of animal fat was negatively associated with *f*_PUFA_ in SAT, resulting in a weak correlation (R = – 0.43, p = 0.010). The systolic blood pressure was negatively associated with *f*_PUFA_ in VAT, but with a weak correlation (R = – 0.44, p = 0.009). Regression coefficients (Beta) and p-values for all significant associations are listed in Table 4. The linear regression model did not detect any statistically significant associations between FAC and age, anthropometric measures, or blood sample analyses.

**Table 4.** Associations between fractions of saturated, monounsaturated, and polyunsaturated fatty acids (f_SFA_, f_MUFA_, f_PUFA_) and the parameters listed in Table 1 (anthropometric measures, blood pressure, blood sample analyses, and dietary habits), obtained from a multiple linear regression model. Only predictor variables with significant contributions to the linear model are listed

Response variable	R^2^	p-value (vs. constant model)	Predictor variables	Beta	p-value
*f_SFA_* (SAT)	0.46	<0.001	Diet score Animal fat	0.48 3.16	0.002 <0.001
*f_MUFA_* (SAT)	0.11	0.039	Unhealthy diet	0.37	0.039
*f_PUFA_* (SAT)	0.18	0.010	Animal fat	–0.44	0.010
*f_SFA_* (VAT)	0.30	0.003	Diet score Animal fat	0.40 2.79	0.021 0.007
*f_MUFA_* (VAT)	0.19	0.010	Unhealthy diet	0.47	0.010
*f_PUFA_* (VAT)	0.19	0.010	Systolic blood pressure	–0.46	0.010

**Figure 4.**
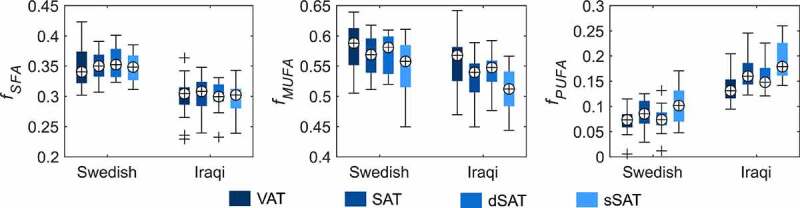
Boxplot of the estimated fSFA, fMUFA, and fPUFA of VAT, SAT, sSAT, and dSAT. Significantly lower fSFA and higher fPUFA were found in all the investigated adipose tissue depots of Iraqi-born men compared to the corresponding depot of Swedish-born men. In the case of fMUFA, lower relative amounts were found in SAT and sSAT of the Iraqi-born men while no difference was found in VAT and dSAT. Comparing VAT and SAT instead, no differences were found between VAT and SAT of Swedish-born men while among the Iraqi-born men, significant differences were found in all parameters except fSFA. In the case of comparing sSAT and dSAT, significant differences were found in parameters of both subject groups except for fSFA among the Iraqi-born men.

Representative examples of the calculated *f_SFA_,f_MUFA_*, and *f_PUFA_* within the subcutaneous and visceral ROIs of an Iraqi-born man and a Swedish-born man are depicted in Figure 4. Differences between the two subjects are visible, especially in the *f_SFA_* and *f_PUFA_* maps. While the *f_SFA_* maps are relatively homogeneous, a spatial, likely artefactual, variation from right to left, along the frequency encoding direction, can be noted in the *f_MUFA_* and *f_PUFA_* maps.

**Figure 5.**
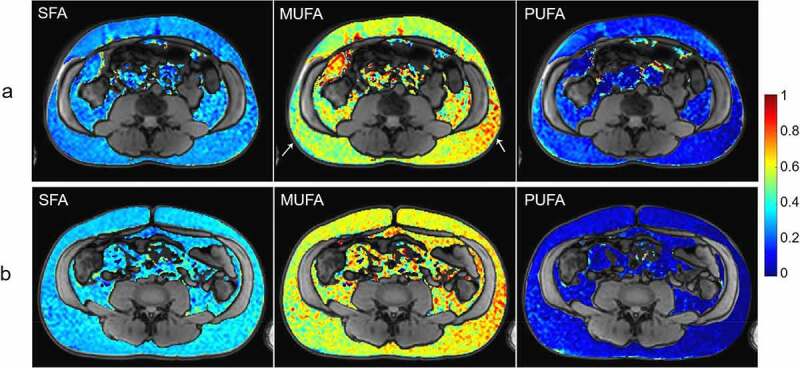
Examples of estimated fSFA, fMUFA, and fPUFA maps of a) an Iraqi-born and b) a Swedish-born man. Especially the fSFA and fPUFA are visibly different in the two persons. The estimated maps have been masked so that only voxels within the subcutaneous and visceral masks (Figure 2), with fat fraction between 0.9 and 1.1, and T2* >20 ms are shown. The two white arrows mark areas where a, presumably artefactual, spatial gradient is visible in the frequency encoding direction.

For the FAC of the adipose tissue depots within each subject group, significantly higher *f_MUFA_* (p < 0.001) and lower *f_PUFA_* (p < 0.001) were found in the VAT compared to the SAT of the Iraqi-born men while no significant difference was found in *f_SFA_* (p = 0.5). In contrast, no significant difference in *f_SFA_,f_MUFA_*, or *f_PUFA_* between VAT and SAT could be found among Swedish-born men (p = 1, p = 0.06, and p = 0.12, respectively). Further, significant differences were found between the dSAT and sSAT depots among both the Iraqi-born and Swedish-born men. A higher *f_SFA_* and *f_MUFA_*, and a lower *f_PUFA_* (p = 0.02, p = 0.02, and p = 0.002, respectively) were found in dSAT among the Swedish-born men. Similarly, higher *f_MUFA_* and lower *f_PUFA_* (p < 0.001 in both cases) were found in dSAT among the Iraqi-born men. However, no difference in *f_SFA_* (p = 0.9) could be found.

Similar to the comparisons of saturation fractions, the Iraqi-born men had significantly higher *ndb* and *nmidb* values than Swedish-born men in all examined adipose tissue depots (SAT, VAT, sSAT, and dSAT), that is, the Iraqi group had a generally less saturated FAC. Comparing instead the different depots, significant differences were found between VAT and SAT (p = 0.001 and p < 0.001 for *ndb* and *nmidb*, respectively), and between dSAT and sSAT (p < 0.001 in both cases) of the Iraqi-born men. Among the Swedish-born men, no difference in FAC of VAT or SAT (p = 0.2 and p = 0.1 for *ndb* and *nmidb*, respectively) was found while significantly different *ndb* (p = 0.02) and *nmidb* (p = 0.02) were found between dSAT and sSAT. To summarize, VAT was significantly more saturated than SAT in the Iraqi-born group, while this was a non-significant tendency among Swedish-born. Similarly, dSAT was significantly more saturated than sSAT, both in Iraqi-born and Swedish-born.

## Discussion

This is one of the first studies to estimate FAC of subcutaneous and visceral adipose tissue in people of different ethnicities. In this study, assessing FAC with an MRI-based method, Iraqi men were found to have a healthier profile with more unsaturated fatty acids in all depots than Swedish men. Further, SAT was less saturated than VAT only in the Iraqi men. These results show that this MRI technique can be used to investigate differences in FAC between different adipose tissue depots, as well as different populations.

The estimated *f_SFA_,f_MUFA_*, and *f_PUFA_* were in good agreement with the typical ranges presented in previous studies where FAC of SAT has been assessed with various methods. Studies using gas chromatography reported *f_SFA_,f_MUFA_*, and *f_PUFA_* within the ranges 0.24–0.37, 0.44–0.63, and 0.12–0.19 [9,24,27,36], while estimations by MRI or MRS were within the ranges 0.29–0.38, 0.45–0.65, and 0.06–0.16, respectively [24,27]. In agreement with our study, several previous studies comparing FAC of different adipose tissue depots have shown that VAT holds a larger proportion of saturated fatty acids than SAT [40,41]. Similar results are reported comparing dSAT and sSAT, with a higher relative amount of saturated fatty acids in dSAT [16,17].

In a previous method validation study, we found good agreement between FAC obtained by MRI and by gas chromatography in the SAT of leg oedema patients [27]. Although it is difficult to directly compare results from studies using different methods, or on different patient groups, the fact that the results presented in this study are consistent with previously reported data, further supports that the MRI-based approach described in the present study is a feasible method for FAC quantification. In addition to MRI being more convenient compared to the invasive gas chromatography approach, it also offers a more objective and operator-independent approach compared to MRS-based techniques. Further, due to differences in chemical shift, the fat and water signals are assigned to different physical locations of the MRS voxel, which may be centimetres apart. This displacement of fat is a much smaller problem for MRI, where the displacement typically can be on a sub-millimetre scale.

Investigations of adipose tissue FAC across healthy people of different ethnicities are rare. A pilot study found lower fractions of MUFA in south Asians than in Latin Americans and north Europeans, but no differences in SFA and PUFA [42]. A more recent study of the plasma metabolic profile of Iraqi-born men and women in Sweden reported lower amounts of SFA (12:0, 14:0, and 16:0) and MUFA (18:1), and a higher amount of PUFA (18:2) in plasma of Iraqi-born subjects compared to a Swedish-born population [43], a trend, which was found also in the present study. In our study, the Iraqi men had a lower consumption of food containing animal fat. This indicates a healthier fat consumption with a lower intake of saturated fat that can contribute to our findings. Indeed, we found that frequent consumption of animal fat was negatively correlated to *f*_PUFA_ and positively correlated to *f*_SFA_. Given the known higher risk of developing type 2 diabetes in Middle Eastern immigrants, our findings are somewhat contradictory. Still, previous population-based studies have reported that the Iraqi-born population has lower blood pressure and better kidney function than Europeans, and seems protected from mortality in cardiometabolic diseases [3,44]. The differences could pinpoint additional risk factors contributing to onset of type 2 diabetes, or be due to the fact that the subjects in the current study are healthy and do not display a change in FAC that could be described as a risk factor at this time point.

More favourable blood pressure levels are speculated to be related to differences in diet [43], and consumption of dietary MUFA and PUFA may have a positive effect on cardiovascular health, in contrast to dietary SFA that is suggested to have a negative impact [12,45]. For instance, the Mediterranean diet, which consists of relatively high amounts of unsaturated fats compared to saturated fats, has been shown to decrease the mortality and morbidity of CVD as well as reduce the risk of recurring cardiovascular events [46]. The diet may be reflected particularly in the *f_PUFA_*, since the essential fatty acids are polyunsaturated [47]. Therefore, the healthier dietary habits and lower systolic blood pressure of the Iraqi-born men seem to support the finding of a negative association between systolic blood pressure and *f_PUFA_* in VAT. Together, the results of this study may reflect a cardiovascular favourable lipid profile that can contribute to the lower risk of atherosclerosis and lower risk of hypertension previously reported among Iraqi-born men and women in Sweden [3].

The differences in FAC between the adipose tissue in the visceral and subcutaneous depots as well as between dSAT and sSAT found in this study are supported by previous studies [15,16,48]. Although the exact mechanism leading to FAC differences, and the role it may have in the development of metabolic or cardiovascular conditions are not known, it has been suggested that VAT and SAT have different metabolic functions [49] and that VAT therefore has a larger role in the development of metabolic diseases [6,7]. Indeed, a limited expansion of the SAT is considered to drive ectopic lipid deposition in other organs, which contributes to metabolic disease. Also, dSAT is proposed to associate with VAT and therefore has a greater significance in the development of various diseases compared to sSAT [50]. The reported differences in FAC and the distinct metabolic roles of various adipose tissue depots, highlights the need of further studies to detangle how FAC and adipose tissue contribute to onset of metabolic diseases. The present study illustrates the use of MRI for such analyses, offering a non-invasive and image-based technique for FAC quantification.

The main limitation of the study is the small number of participants. With more participants, other factors that can affect the FAC in adipose tissue, such as physical activity [51], could have been investigated. However, the groups were well matched regarding age, anthropometrics and metabolism, and our data could still show significant differences in MRI assessed FAC across men of Middle East and European ancestry. Methodologically, a possible source of bias is the observed spatial variation in the estimated FAC maps along the readout (left-right) direction. This, likely artefactual, variation has been described previously [20], but is, however, not expected to have a great impact on the comparisons as the artefact affects the FAC estimations of both subject groups in a similar way. Although the current MRI technique cannot provide detailed information about individual fatty acids, as opposed to gas chromatography, it offers a non-invasive method to assess spatial variations of FAC. However, separation of omega-3 from omega-6 has been possible using MRS [52]. Future developments of acquisition protocols and reconstruction algorithms may allow this separation also by MRI.

### Conclusion

Using MRI, different FAC were found in the abdominal region of healthy Iraqi-born men as compared to Swedish-born men, independently of age and BMI. The Iraqi-born men had a higher fraction of PUFA and lower fractions of SFA and MUFA, in both VAT and SAT. The more unsaturated FAC of the adipose tissue in Iraqi healthy men may indicate that this population has a favourable phenotype for cardiometabolic disease including obesity and type 2 diabetes, protecting them from future complications, such as hypertension, chronic kidney disease and mortality, but this hypothesis needs to be further investigated.

This study provided valuable information, both in respect to verification of the MRI-method itself, as well as providing novel knowledge that is valuable to resolve different risk factors behind metabolic disease related to ethnicity.

## Data Availability

All data acquired and analyzed during the present study are not publicly available, but are available from the corresponding author on reasonable request.
